# Major cardiovascular events and associated factors among routine hemodialysis patients with end-stage renal disease at tertiary care hospital in Somalia

**DOI:** 10.3389/fmed.2023.1086359

**Published:** 2023-05-19

**Authors:** Mohamed Farah Yusuf Mohamud, Faduma Nur Adan, Mohamed Osman Omar Jeele, Mohamed A. M. Ahmed

**Affiliations:** ^1^Mogadishu Somali Turkish Training and Research Hospital, Mogadishu, Somalia; ^2^Department of Pediatric, Mogadishu University, Mogadishu, Somalia

**Keywords:** hemodialysis, end-stage renal disease, cardiovascular disease, heart failure, diabetic, hypertension

## Abstract

**Introduction:**

Cardiovascular complications are the most significant cause of death in patients undergoing routine hemodialysi (HD) with end-stage renal disease (ESRD). The main objective of this study is to determine the significant cardiac events and risk factors in patients undergoing routine hemodialysis in Somalia.

**Methods:**

We carried out a cross-sectional retrospective study in a single dialysis center in Somalia. Two hundred out of 224 were included. All of them had ESRD and were on hemodialysis during the study period between May and October 2021. The records of all patients were reviewed, and the following parameters were analyzed socio-demographic factors, risk factors for cardiovascular disease, and the presence of cardiovascular diseases.

**Results:**

The mean age was 54 ± 17.5 years (range 18–88 years), and 106 (53%) patients were males. The prevalence of a cardiovascular disease among hemodialysis patients was 29.5%. Moreover, the distribution of cardiovascular diseases was different; heart failure was the most common, about 27.1%, followed by coronary artery disease (17%), pericarditis and pericardial-effusion (13.6%), dysrhythmia (10.2%), cerebrovascular-accident (8.5%), and peripheral vascular disease (3.4%). About 176 (88%) participants had at least one modifiable cardiovascular risk factor. The most common modifiable cardiovascular risk factor was hypertension (*n* = 45, 25.1%), followed by anemia (*n* = 28, 15.6%) and diabetes (*n* = 26, 14.5%). Younger (18–30) participants were six times less likely to have cardiovascular events among hemodialysis than older age 0.4 (0.11–1.12).

**Conclusion:**

Low prevalence rate of cardiovascular complications was confirmed in ESRD patients receiving hemodialysis in the main HD center in Somalia. Diabetes, anemia, and hypertension were the highest significant risk factors for CVD in HD patients with ESRD in Somalia.

## Introduction

CKD is becoming more common in Sub-Saharan Africa (SSA), primarily affecting young individuals in their prime for economic productivity. Additionally, many patients receive nephrologist referrals too late, are vulnerable to acute complications from dialysis, and struggle with infrastructure and financial issues that make it challenging to provide adequate dialysis to those with end-stage renal disease (1, 2). Although the prevalence of chronic kidney disease (CKD) in Somalia has not previously been studied, Muiru et al. reported that CKD in sub-Saharan Africa was determined to be 8% in 2020 (3).

Renal replacement therapy (RRT) as a whole and hemodialysis (HD) in particular remains a lifesaving intervention for a lot of patients whose kidneys do not work correctly (4). Nevertheless, in patients undergoing routine hemodialysis (HD), there is an increased risk for cardiovascular morbidity and mortality (5).

It's thought that cardiovascular diseases account for more than 50% of deaths among HD patients (6). Cardiovascular deaths among HD patients are also believed to be 10–20 times greater than in general (7). Sudden cardiac death is the leading cardiovascular death among HD patients, with 25% of all cardiovascular deaths (8).

Some researchers suggested that the HD process can activate the complement system, induce prothrombotic and proinflammatory responses in patients and thus predispose cardiovascular events to the development (9). Some researchers suggest that cardiovascular lesions may even appear before the initiation of the HD process in chronic renal failure patients, as chronic renal failure or end-stage renal disease (ESRD) is an independent risk factor for cardiovascular diseases (10). In addition, HD can lead to anemia and cause alteration in calcium and phosphate metabolism, which could play a significant risk factor for developing cardiovascular events (11).

To the best of our knowledge, the prevalence and risk factors of major cardiac events among patients undergoing HD in Somalia remain unknown. The main objective of this study is to determine the significant cardiac events and risk factors among patients undergoing routine hemodialysis in Somalia.

## Methods

This retrospective study included all patients who have received the diagnostic code of ESRD in accordance with the International Classification of Diseases (ICD-10) system and underwent routine hemodialysis between May 2021 and October 2021 using the electronic hospital information system (HIS). Two hundred out of 224 patients who had ESRD and underwent routine hemodialysis (HD) were included in our study. Patients with renal transplantation, peritoneal dialysis, and those with incomplete data were excluded from the study.

The following parameters were analyzed: Socio-demographic and clinical parameters included age and gender, risk factors for cardiovascular disease (family history of coronary artery disease, diabetes mellitus, arterial hypertension, and dyslipidemia), anemia, duration on HD, and time of hemodialysis per week. The presence of cardiovascular diseases such as heart failure, coronary artery disease, dysrhythmia, pericarditis, pericardial effusion, cerebrovascular disease, and peripheral vascular disease was looked for in the hospital information system (FONET) record by using an electrocardiogram, two-dimensional and Doppler echocardiography, Doppler ultrasound, brain CT scan, and brain MRI records.

The Echocardiography was licensed in Turkey using a Toshiba Aplio™ ultrasound system (TUS-A500, Shimoishigami, Japan) in accordance with the American Society of Echocardiography guidelines.

The presence of any of the following was considered a cardiovascular disease (CVD):

- Coronary heart disease: myocardial infarction or stable angina or unstable angina by assessing normal values for cardiac dimensions and EKG diagnostic criteria were obtained from standard references, or coronary artery bypasses graft or percutaneous coronary intervention (12).
- Heart failure: is defined as an aberrant left ventricular filling pattern and/or a mitral E/A ratio on echocardiography that is out of the range of 0.7–3.1 if under 64 years old or 0.5–1.7 if over 64 years old. b) Systolic dysfunction was defined as an ejection fraction of <50%.
- Cerebrovascular disease includes atherothrombotic cerebral infarction and transient ischemic attack with brain CT scan or MRI Confirmation.

- Peripheral vascular disease: is a chronic progressive atherosclerotic disease leading to partial or total peripheral vascular occlusion. PAD typically affects the abdominal aorta, iliac arteries, lower limbs, and occasionally the upper extremities.
- Dysrhythmias: evidence of ventricular tachycardia, fibrillation, or any other type of dysrhythmia on electrocardiographic criteria.
- Pericarditis and Pericardial effusion: diffuse ST-segment elevation on ECG, stiff or thick pericardium constricting the heart's normal movement or free fluid around the heart by echocardiography.

The study was carried out after receiving ethical approval and being granted permission by the research and ethical committee of the Mogadishu Somali Turkish Training and Research Hospital (Ref: MSTH/6384). This study was carried out in accordance with the Helsinki Declaration's contents. The information obtained from the medical records was kept strictly confidential and utilized only for research purposes. Furthermore, study participants are not recognized by name to ensure confidentiality.

Microsoft Excel and SPSS software version 23 were used to create the database. Continuous variables are presented as mean ± standard deviation, and categorical variables as the observed number of patients (percentage). Fisher's exact test was used for categorical variables to compare patient characteristics between groups (cardiac and non-cardiac events). For correlations, a correlation coefficient test was applied, binary logistic regression was also used, and a *p*-value of < 0.05 was considered statistically significant.

## Results

In this retrospective observational study, 200 out of 224 routine hemodialysis patients at Mogadishu Somali Turkish Training and Research Hospital from May 1, 2021, to October 231, 2021, fulfilled the inclusion criteria and enrolled in the study.

Table 1 shows the socio-demographic characteristics of the 200 patients with HD with ESRD. The mean age was 54 ± 17.5 years (range 18–88 years), and 106 (53%) patients were males.

**Table 1:** Socio-demographic characteristics among hemodialysis patients with end-stage renal disease (*N*: 200).

**Variables**		**All (*n* = 2) Freg (%)**	**Cardiac events**		***P*-value**
			**Yes**, ***N*** = **59**	**No**, ***N*** = **141**	
Age group	18–30 years	30 (15%)	10 (17%)	20 (14.2%)	0.086
	31–49 years	39 (19.5%)	6 (10.2%)	33 (23.4%)	
	50–69 years	89 (44.5%)	26 (44%)	63 (31.5%)	
	≥70 years	42 (21%)	17 (28.8%)	25 (12.5%)	
Sex	Male	106 (53%)	30 (50.8%)	76 (53.9%)	0.757
	Female	94 (47%)	29 (49.2%)	65 (46.1%)	
Time of hemodialysis	One a week	29 (14.5%)	5 (8.5%)	24 (17%)	0.259
	Twice a week	156 (78%)	50 (84.7%)	106 (75.2%)	
	Three times a week	15 (7.5%)	4 (6.8%)	11 (7.8%)	
Duration of hemodialysis	≤ 1 year	62 (31%)	23 (39%)	39 (27.6%)	0.222
	2–5 years	77 (38.5%)	22 (37.3%)	55 (39%)	
	>5 years	61 (30.5%)	14 (23.7%)	47 (33.3%)	
Risk factors	Yes	176 (88%)	56 (95%)	120 (85.1%)	0.135
	No	22 (12%)	3 (5%)	21 (14.9%)	

Based on the time of hemodialysis, most of the patients (78%) underwent hemodialysis twice a week. In comparison, 29 (14.5%) patients underwent one a week, and 15 (7.5%) patients underwent three times per week.

According to the hemodialysis duration, most patients (38.5%) experience hemodialysis for 1–5 years. Notably, the duration of hemodialysis < 1 year and >5 years were less, being experienced in 31 and 30.5%, respectively (*P* = 0.222) (Table 1).

**Figure 1:**
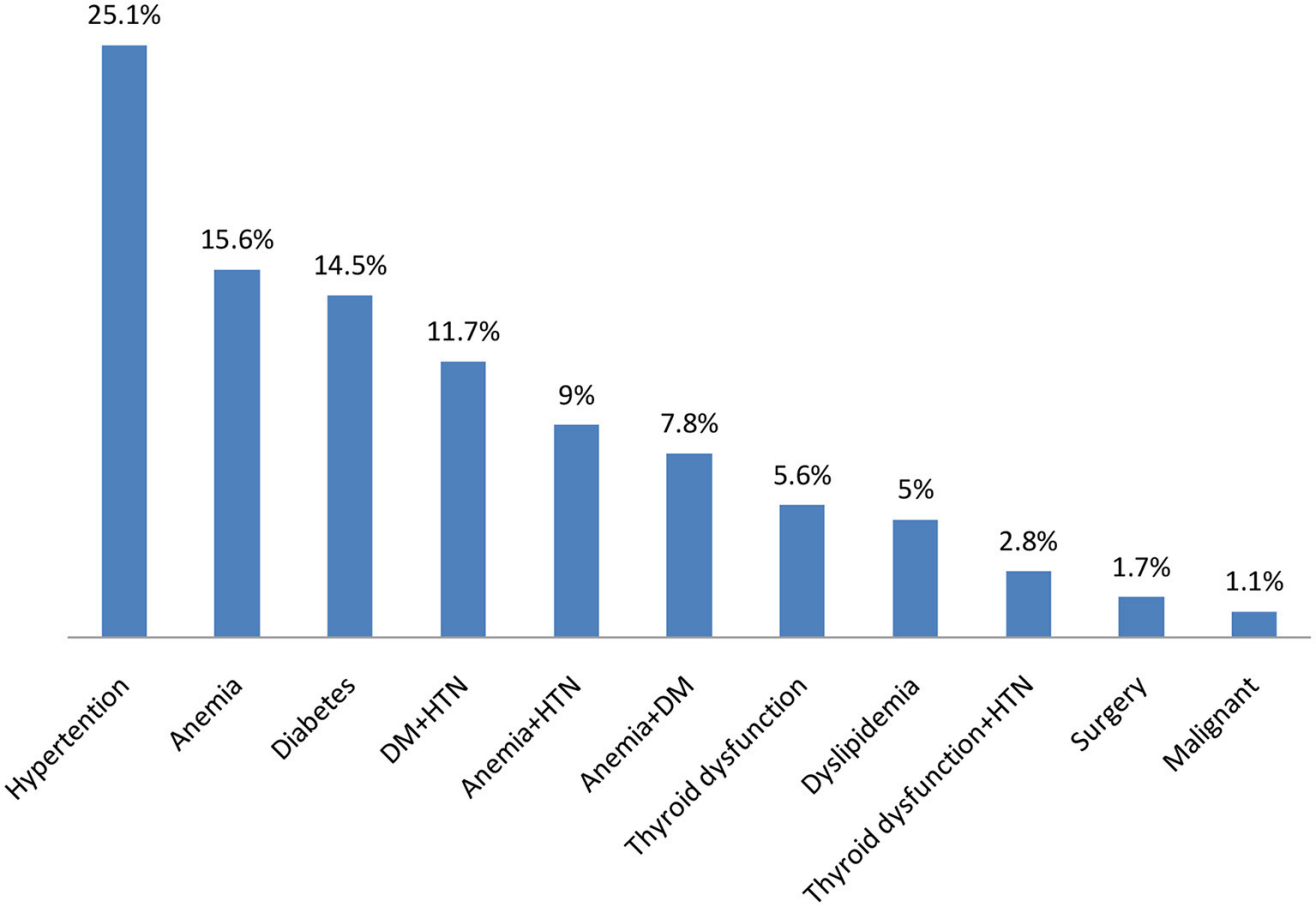
Cardiovascular risk factors among hemodialysis patients with ESRD in Somalia. DM, Diabetes; HTN, Hypertension.

This study revealed that about 176 (88%) of the study participants (hemodialysis patients with end-stage renal disease) had at least one modifiable cardiovascular risk factor. As shown in Figure 1, the most common modifiable cardiovascular risk factors among hemodialysis patients with end-stage renal disease were hypertension in 45 patients (25.1%), followed by anemia in 28 patients (15.6%), and diabetes mellitus in 26 patients (14.5%).

**Figure 2:**
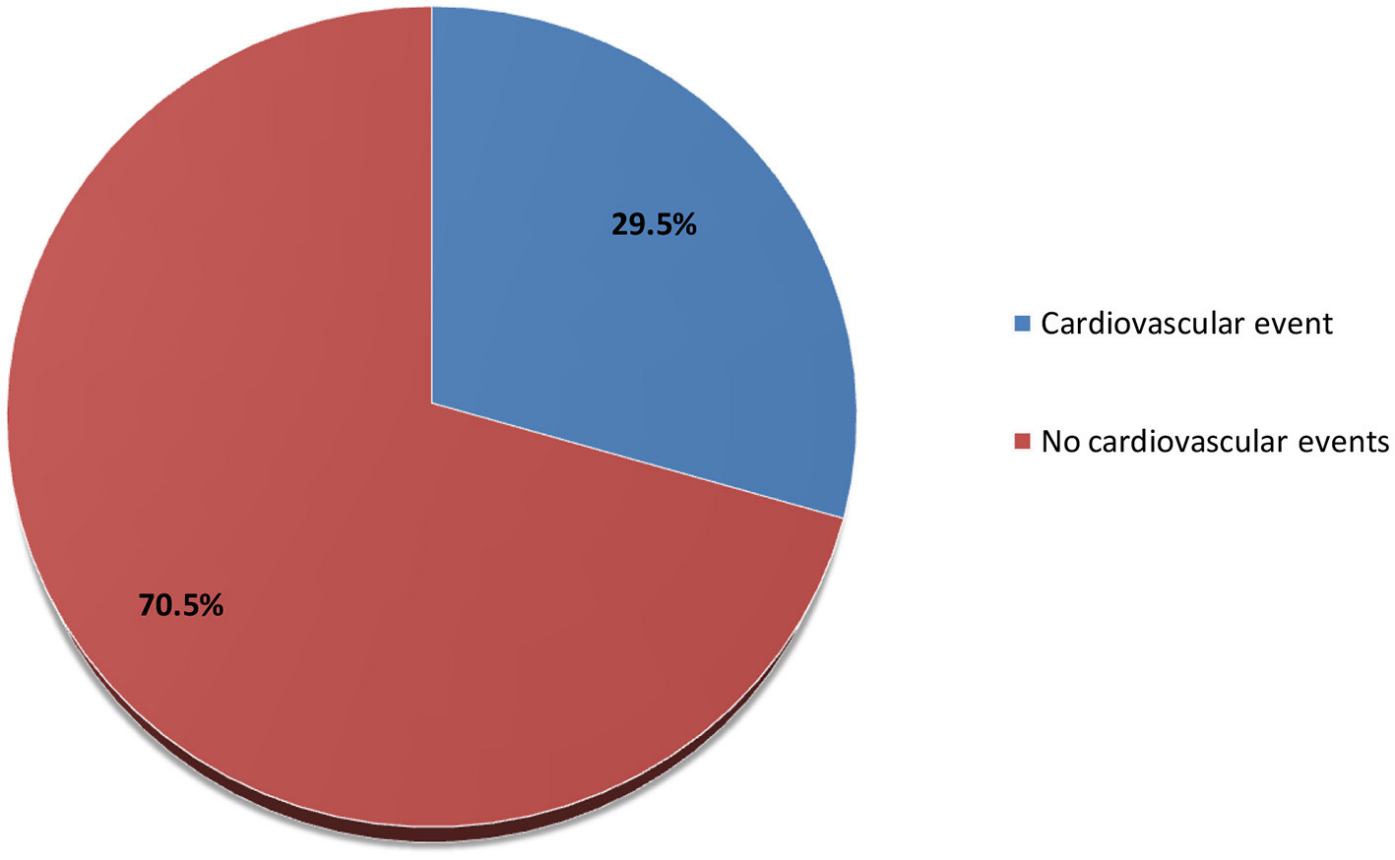
Prevalence of cardiovascular disease among hemodialysis patients with ESRD in Somalia.

**Figure 3:**
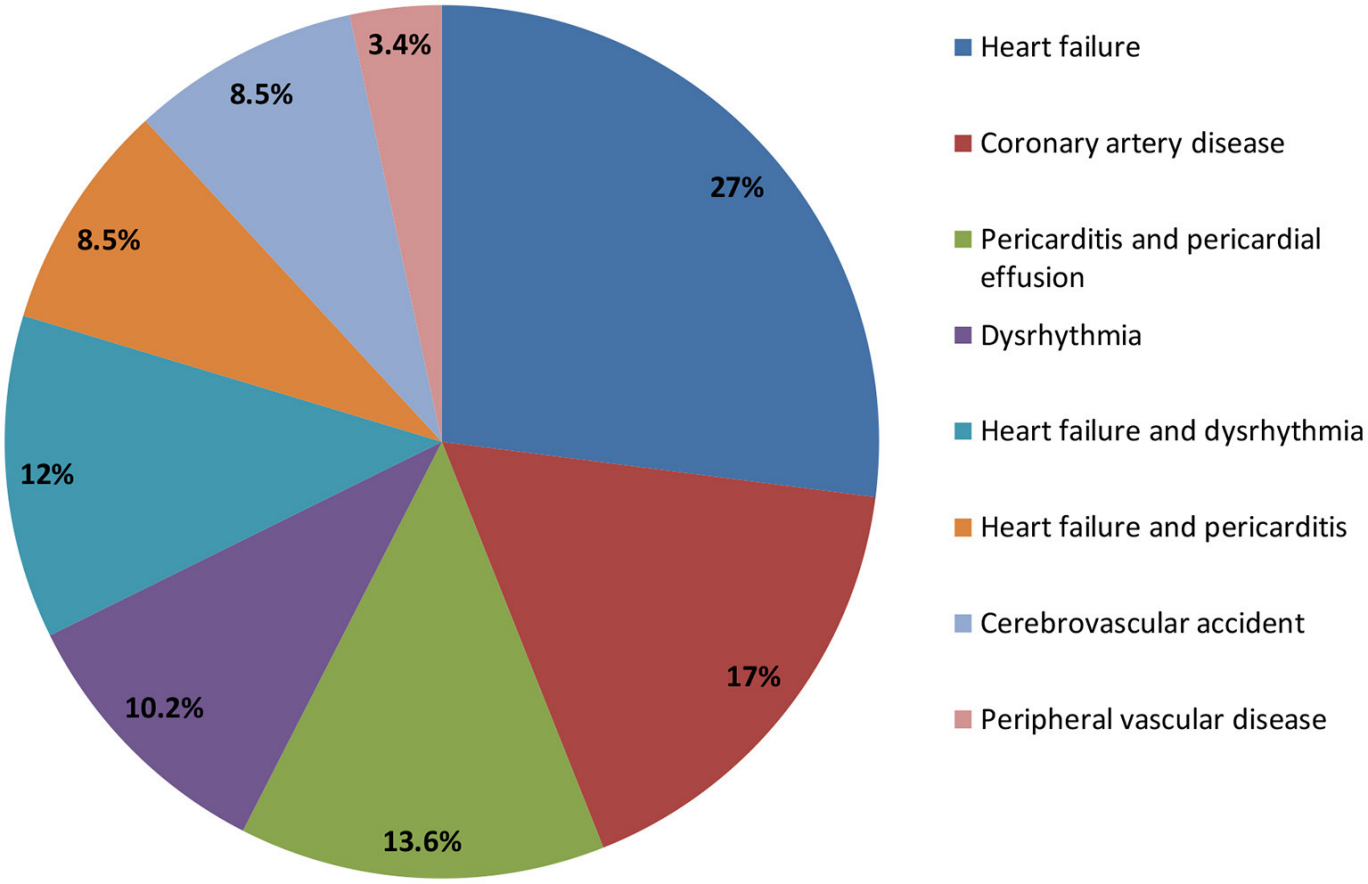
Distribution of cardiovascular events among hemodialysis patients with ESRD in Somalia.

The prevalence of cardiovascular disease among hemodialysis patients with ESRD was 29.5%, as shown in Figure 2. About 27.1% of the hemodialysis patients with ESRD had heart failure, 17% had coronary artery disease, 13.6% had pericarditis and pericardial effusion, 10.2% had dysrhythmia, 8.5% had cerebrovascular accident, and 3.4% had peripheral vascular disease (Figure 3).

The data also showed among the 59 respondents that were diagnosed with at least one cardiovascular disease, near half of the respondents [30 out of 59 (50.8%)] were males. In comparison, females comprised the remaining 29 out of 59 respondents (49.2%) (*p* = 0.757).

Among the age subgroups, the prevalence of CVD was 16.9, 10.1, 44, and 28.8% in 18–30 years, 31–49 years, 51–69 years, and 70 years or older, respectively (*p* = 0.086).

Of the 16 respondents who were diagnosed with at least one cardiovascular disease, 23 (40%) had been on hemodialysis for 1 year or less, and 22 (37.3%) respondents have on hemodialysis for 2–5 years, while the remaining 14 respondents (23.7%) have been on hemodialysis for more than 5 years (*p* = 0.222).

Regarding the study population, 59 respondents were diagnosed with at least one cardiovascular disease. Most participants (84.7%) had undergone hemodialysis twice per week, while five (8.5%) had undergone hemodialysis once weekly. Only four (6.8%) participants had undergone hemodialysis three times or more per week (*p* = 0.259) (Table 2).

**Table 2:** Age and risk factor differences in prevalence of cardiovascular events among routine hemodialysis patients with ESRD.

**Variable**	**Odds ratio**	**95% CI**	***p*-value**
**Age group**			
18–30 years	1.0
31–49 years	0.4	0.11–1.12	0.086
50–69 years	0.8	0.34–2.00	0.671
≥70 years	1.4	0.51–3.62	0.538
**Risk factors**			
Yes	1.0
No	0.3	0.09–1.21	0.096

Table 2 shows younger (18–30) participants were six times less likely to have cardiovascular events among hemodialysis than older age 0.4 (0.11–1.12). Cardiovascular events were less in participants with previous risk factors than in those without.

## Discussion

Chronic kidney disease (CKD) implies various degrees of declined renal function. The most severe and last stage of CKD is an end-stage renal disease (ESRD), which occurs when the kidneys cannot properly perform their essential functions. Finding regular hemodialysis or a kidney transplant is the only option available to individuals with ESRD to survive (13, 14).

Cardiovascular complications are the most significant cause of death in patients with end-stage renal disease (ESRD) on hemodialysis treatment (15). As early as 1836, Richard Bright suggested that the first cardiovascular disease (CVD) originated from renal disease (16).

The mechanism underlying the increased risk of cardiovascular events in patients with ESRD has not been well defined. In fact, a broad spectrum of risk factors influences cardiac function and structure in hemodialysis patients with ESRD.

Lindner et al. (17) discovered the significant burden of cardiovascular disease (CVD) in chronic renal disease (CRD) more than 40 years ago.

In general medical practice, patients in stages 3–4 CKD who have reduced renal function but are not in ESRD have a prevalence of ischemic heart disease of 25%, more than double the prevalence in patients without CKD, according to the NEORICA (New Opportunities for Early Renal Interventions by Computerized Assessment) study (18).

Cardiovascular disease is a common cause of death in hemodialysis patients, with a ratio of 10–20 times greater than in people with normal renal function (19).

Regarding the duration of HD, most patients (38.5%) had dialysis duration between 1 and 5 years, similar to study findings from Sudan (20).

Our findings showed that most of the participants (84.7%) had undergone hemodialysis twice per week, while five (8.5%) participants had undergone hemodialysis once per week. Only four (6.8%) participants had undergone hemodialysis three times or more weekly. In contrast to our report, a study from Ethiopia found that only 10.8% had undergone hemodialysis twice per week, while 89.2% had undergone hemodialysis Three times per week (21).

Our study found no association between cardiovascular disease and duration of HD and section of HD per week.

A hemodialysis study in the United States states that 40% of dialysis patients had cardiovascular disease at admission. Coronary artery disease was the cause of 63% of hospital admissions for cardiovascular causes (22).

In the present study, the prevalence of cardiovascular disease among hemodialysis patients with ESRD was 29.5% lower than that reported in previous studies (22, 23). Another study from Cameroon found that 84% of hemodialysis patients with ESRD had a cardiovascular illness, which is higher prevalent than this figure (2). The variation in cardiovascular events prevalence in our study compared with other reports could be due to several reasons: the diagnostic criteria for cardiovascular events in ESRD patients were not uniform, the different populations were genetically varied, or the inclusion criteria in the various studies may have been different.

The distribution of cardiovascular diseases among hemodialysis patients with ESRD was different. In our study, heart failure (27.1%) was the most common cardiovascular disease among hemodialysis patients with ESRD, followed by coronary artery disease (17%), pericarditis and pericardial effusion (13.6%), dysrhythmia (10.2%%), cerebrovascular accident (8.5%), and peripheral vascular disease (3.4%).

A randomized multi-center trial on 1,846 chronic hemodialysis patients at 15 clinical centers comprising 72 dialysis units, congestive heart failure (40%), ischemic heart disease (IHD) (39%), and arrhythmia (31%) were the most common prevalent cardiovascular disease (22).

Analysis of the Yaoundé General Hospital (YGH) hemodialysis center from December 2010 to February 2011 revealed that left ventricular hypertrophy (60%), valvular calcifications (38%), heart failure (36%), conduction disorders (33%), pericardial effusion (13%), valvular diseases (11%) and ischemic heart diseases (2%) were the highest distribution of cardiovascular diseases among hemodialysis patients with ESRD (2).

The increased prevalence of heart failure could be related to the higher prevalence of hypertension in this study sample which is the leading etiological factor of underlying renal disease. Another risk factor for heart failure is the high incidence of anemia caused by the low use of erythropoiesis-stimulating drugs. High cardiac output, a large stroke volume, increased heart rate, and deteriorating left ventricular dilatation are all associated with anemia (24).

A study from Spain on cardiovascular disease among hemodialysis patients reported that 16.7% had coronary disease, 13.9% had different degrees of heart failure, and 11.6% had arrhythmia (25). Rostand and his teammates reported that 73% of hemodialysis patients have coronary artery disease, representing the highest cardiovascular disease prevalence among ESRD patients (26).

In our study, 88% of dialysis patients had at least one pre-existing comorbidity before beginning dialysis therapy, significantly higher than the study published in Malaysia, which found that just 31.6% had such conditions (23). The most prevalent comorbidities in the current study were hypertension (21.5%), anemia (15.6%), and diabetes (14.4%). In bivariate or multivariate analyses, pre-existing comorbidities were also not statistically related to cardiovascular events. According to Lim et al. (23), hypertension (96.5% of all cases), diabetes (66.2%), and hyperlipidemia (58.1%) were the most prevalent comorbidities identified throughout their analysis.

Cardiovascular events were less in participants with previous risk factors than in those without. This may be due to progress in both prevention and treatment of CVD, including precipitous declines in cigarette smoking, improvements in hypertension and diabetic treatment and control, and widespread use of statins to lower circulating cholesterol levels.

Regarding the study population, most of the participants (84.7%) had undergone hemodialysis twice per week, while five (8.5%) participants had undergone hemodialysis once per week. Only four (6.8%) participants had undergone hemodialysis three times or more per week (*p* = 0.259). Inadequate or missed hemodialysis sessions also significantly affected cardiovascular disease among hemodialysis patients with ESRD. The overcrowding of our center for receiving many ESRD patients needing regular renal replacement therapy, lack of public awareness of the disease and the hemodialysis itself, discrimination, and social pressure on the patients were the leading factors of inadequate or missed hemodialysis sessions. In addition to this, insufficient skills of dialysis providers, higher costs belonging to each dialysis session that most of the patients are not affordable (low socioeconomic status), as well as; lack of access to the center because of rural and far distance distribution of the cases also played a role.

The limitations of our study included: a limited sample size, a retrospective study, and a single-center study that may not be representative of the country. Risk factors such as smoking, alcoholism, sedentary lifestyle, and obesity were not evaluated due to a retrospective study that cannot be obtained from the system. Several novel risk factors have yet to be explored due to technical drawbacks and the high cost of laboratory-based tests.

Despite the growing population of patients on maintenance hemodialysis in Somalia, there has been a relative lack of large clinical databases describing the specific cardiac diseases among HD patients with ESRD.

Although this study has several limitations, it is the first study to assess the prevalence, risk factors, and extent of cardiovascular disease in a significant condition in adult chronic hemodialysis patients in Somalia. This issue has been well addressed in adult ESRD patients.

## Conclusion

Cardiovascular events are lower prevalent among hemodialysis patients with ESRD in Somalia when compared to other countries. The majority of the cardiovascular event confirmed in our HD patients were significantly higher in older patients and those with diabetes, anemia, and hypertension.

## Data availability statement

We declared that we had full access to all of the data in this study and we take complete responsibility for the integrity of the data. All original data are available in the Mogadishu Somali Turkish Training and Research Hospital in Mogadishu, Somalia. Data used to support the findings of this study are available from the corresponding author upon request.

## Ethics statement

The study was carried out after receiving ethical approval and granted permission from the Research and Ethical Committee of the Mogadishu Somali Turkish Training and Research Hospital (Ref: MSTH/6384). Written informed consent for participation was not required for this study in accordance with the National Legislation and the Institutional requirements.

## Author contributions

All authors made a significant contribution to the work reported, whether that is in the conception, study design, execution, acquisition of data, analysis and interpretation, or in all these areas, took part in drafting, revising or critically reviewing the article, gave final approval of the version to be published, have agreed on the journal to which the article has been submitted, and agree to be accountable for all aspects of the work.
